# Comprehensive Analysis to Identify LINC00511–hsa-miR-625-5p–SEMA6A Pathway Fuels Progression of Skin Cutaneous Melanoma

**DOI:** 10.1155/2023/6422941

**Published:** 2023-07-03

**Authors:** Guanghua Chen, Jia Yan, Zhou Fu

**Affiliations:** ^1^Department of Dermatology, Children's Hospital of Chongqing Medical University, National Clinical Research Center for Child Health and Disorders, Ministry of Education Key Laboratory of Child Development and Disorders, Chongqing Key Laboratory of Child Infection and Immunity, Chongqing 400014, China; ^2^Department of General Surgery, University-Town Hospital of Chongqing Medical University, Chongqing 401331, China; ^3^Department of Respiratory, Children's Hospital of Chongqing Medical University, National Clinical Research Center for Child Health and Disorders, Ministry of Education Key Laboratory of Child Development and Disorders, Chongqing Key Laboratory of Child Infection and Immunity, Chongqing 400014, China

## Abstract

**Objective:**

Skin cutaneous melanoma (SKCM) is a highly lethal malignancy that poses a significant threat to human health. Recent research has shown that competing endogenous RNA (ceRNA) regulatory networks play a critical role in the development and progression of various types of cancer, including SKCM. The objective of this study is to investigate the ceRNA regulatory network associated with the transmembrane protein semaphorin 6A (SEMA6A) and identify the underlying molecular mechanisms involved in SKCM.

**Methods:**

Expression profiles of four RNAs, including pseudogenes, long non-coding RNAs, microRNAs, and mRNAs were obtained from The Cancer Genome Atlas database. The analysis was completed by bioinformatics methods, and the expression levels of the selected genes were verified by cell experiments.

**Results:**

Bioinformatics analysis revealed that the LINC00511–hsa-miR-625-5p–SEMA6A ceRNA network was associated with SKCM prognosis. Furthermore, immune infiltration analysis indicated that the LINC00511–hsa-miR-625-5p–SEMA6A axis may have an impact on changes in the tumor immune microenvironment of SKCM.

**Conclusion:**

The LINC00511–hsa-miR-625-5p–SEMA6A axis could be a promising therapeutic target and a prognostic biomarker for SKCM.

## 1. Introduction

Skin cutaneous melanoma (SKCM) is a deadly form of skin cancer with a high mortality rate compared with other types of skin cancer [1]. According to the 2019 cancer treatment and survival statistics [2], SKCM is among the top five pandemic types of cancer. Early-stage SKCM can be treated with extended resection [3], whereas several patients still develop metastatic tumors with a poor prognosis and a 5-year survival rate of less than 10% [4]. Therefore, it is essential to identify a sensitive and specific biomarker as a therapeutic target and understand the related molecular mechanism for improving the prognosis of patients with SKCM.

Semaphorin 6A (SEMA6A) is a transmembrane-bound protein that plays a role in guiding axons and migrating cells in the nervous system [5]. Recent research has also identified SEMA6A in cancer development [6]. Increased expression of SEMA6A has been reported to be associated with tumor apoptosis in human oral cancer cells [7], whereas low expression of SEMA6A in lung cancer is correlated with high recurrence in the clinical setting [8].

**Figure 1:**
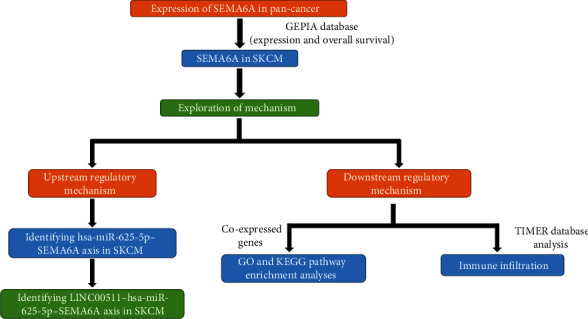
The flow diagram of the study.

The competing endogenous RNA (ceRNA) hypothesis was proposed by Salmena et al. [9], which demonstrated that non-coding RNAs (ncRNAs), such as long ncRNAs (lncRNAs), pseudogenes, and circRNAs, could bind and inactivate microRNAs (miRNAs), ultimately affecting protein-coding by degrading or silencing mRNA [10]. Based on this hypothesis, a ceRNA network related to SKCM progression was developed through a series of analytical procedures (Figure 1).

First, it was attempted to analyze the differential expression level and prognostic risk of SEMA6A in pan-cancer from the Gene Expression Profiling Interactive Analysis (GEPIA) database. By analyzing a variety of tumor tissues, it was found that only SEMA6A, which is highly expressed in SKCM, resulted in reduced overall survival (OS). Next, The Cancer Genome Atlas (TCGA)-SKCM cohort was employed to plot the receiver operating characteristic (ROC) curve to evaluate the specificity and sensitivity of the diagnosis. It was also confirmed that SEMA6A and LINC00511 were highly expressed in skin melanoma cell lines through cell experiments.

Subsequently, the potential downstream modulatory pathways and immune infiltration were explored. Finally, through survival and correlation analysis, the most promising ceRNAs of SEMA6A were identified. The LINC00511–hsa-miR-625-5p–SEMA6A axis provided new insights into the molecular mechanisms of SKCM progression, emerging as an effective therapeutic target and a promising diagnostic biomarker for SKCM.

## 2. Materials and Methods

### 2.1. GEPIA Database Analysis

It was attempted to retrieve data from TCGA and Genotype-Tissue Expression (GTEx) [11, 12] databases to investigate the expression level of SEMA6A in pan-cancer samples. The log2(TPM + 1) score was used to explore the expression level of SEMA6A in different types of cancer. For tumors that had limited normal tissues or non-normal tissues, the “Expression analysis-box plots” and “Survival plots” modules of the GEPIA database were utilized [13]. This enabled us to obtain box plots that compared the expression level of SEMA6A between tumor tissues and normal tissues from the GTEx data. The *p*-value cutoff was set at 0.01, and the log2FC (fold change) cutoff was set at 1 while employing the “Match TCGA normal and GTEx data” option. Furthermore, a survival analysis of SEMA6A was conducted on the high-expression tumor group using the GEPIA database, where the median group cutoff was set at 50% for both Cutoff-High and Cutoff-Low.

### 2.2. Statistical Analysis

A previous study utilized the XIANTAO platform (https://www.xiantao.love/) to evaluate the prognostic values of miRNA and lncRNA in SKCM [14]. The ceRNA hypothesis was followed, where miRNA HR < 1 was indicative of poor OS. To visualize the data, a forest plot was generated using the “ggplot2” R package. In addition, to determine the diagnostic value of SEMA6A expression level in tumor and normal samples of the GTEx database, ROC curve analysis was conducted.

### 2.3. Human Protein Atlas

The Human Protein Atlas (HPA) project is one of the largest biological databases in the world, containing a vast amount of human proteome data and images [15]. For this particular study, an immunohistochemistry image was utilized to directly compare the expression of SEMA6A protein in human skin melanoma tissues versus normal skin tissues.

### 2.4. Human Normal Skin and SKCM Cell Lines

Human primary normal skin melanocytes and SKCM cell lines (A375 and SK-MEL-2) were purchased from Fenghui Biotechnologies Co., Ltd. (Hunan, China). The cells were cultured in a Dulbecco's modified Eagle's medium that was supplemented with 10% fetal bovine serum, 100 U/mL of penicillin, and 100 mg/mL of streptomycin. Following this, the cells were incubated at a temperature of 37°C in an environment containing 5% CO_2_.

### 2.5. Real-Time Quantitative Polymerase Chain Reaction

Based on the manufacturer's instructions, the study involved the extraction of total RNAs from both melanoma cell lines and human primary normal skin melanocyte cell lines using TRIzol (Takara Biomedical Technology (Beijing) Co., Ltd., Beijing, China). Subsequently, cDNA was synthesized by reverse transcription using 1 *μ*g RNA and Oligo-dT Primer (Beijing Liuhe BGI Co., Ltd., Beijing, China). Quantitative polymerase chain reaction (PCR) was performed using the TB-Green method (Takara Biomedical Technology (Beijing) Co., Ltd.) on an Applied Biosystems (ABI) real-time PCR system. The reaction cycle conditions consisted of an initial step at 94°C for 10 minutes, followed by 40 cycles of 95°C for 5 seconds, 60°C for 15 seconds, and 72°C for 10 seconds. The primer sequences were: encoding RNA: SEMA6A forward primer (5′–3′) CCTGCAGCATTTATCACCC, reverse primer (5′–3′) CCATCTGTATTGCCACGCTC and glyceraldehyde-3-phosphate dehydrogenase (GAPDH) forward primer (5′–3′) CTGACTTCAACAGCGACACC, reverse primer (5′–3′) GTGGTCCAGGGGTCTTACTC. ncRNAs: UBQLN4P1 forward primer (5′–3′) GAGATCAGCCACATGCTCAA, reverse primer (5′–3′) AAGGGATTGTTGCCAAACTG and LINC00511 forward primer (5′–3′) CCACAGGAAACCCACACTCT, reverse primer (5′–3′) CACATGGTGGGTAGATGCTG and GAPDH forward primer (5′–3′) AACGGATTTGGTCGTATTGGG, reverse primer (5′–3′) CCTGGAAGATGGTGATGGGAT. Each cDNA sample was measured three times to ensure accuracy.

### 2.6. Co-Expression Gene Analysis of SEMA6A in SKCM

The co-expression of genes associated with SEMA6A expression in SKCM was examined using Pearson's correlation coefficient >0.5 and *p* < 0.05.

### 2.7. Gene Ontology and Kyoto Encyclopedia of Genes and Genomes Pathway Enrichment Analyses

For the analysis and visualization of Gene Ontology (GO) and Kyoto Encyclopedia of Genes and Genomes (KEGG) pathway enrichment analyses of co-expressed genes obtained using Pearson's correlation coefficient >0.5 and *p* < 0.05, the “ClusterProfiler,” “GOplot,” and “ggplot2” R packages were utilized [16, 17]. The assessment of enriched biological processes (BP), molecular functions (MF), and cellular components (CC) for GO analysis was performed.

### 2.8. TIMER Database Analysis

Multiple immune accumulation estimation methods, including XCELL, CIBERSORT, and CIBERSORT-ABS [18–20] were utilized to explore SEMA6A expression level and the abundance of immune infiltrates in cutaneous melanoma. These analyses were performed via the TIMER2 web platform. To query a specific gene, users can click the “Immune Association” button, enter the gene name in the “Gene Expression”: box, and then select relevant immune cells for analysis by clicking on them within the “Immune Infiltrates”: box. B cell naive was selected for further analysis among all the estimated immune cells. The resulting data were presented as heatmaps and scatterplots.

### 2.9. StarBase Database Analysis

The StarBase database primarily concentrates on miRNA-target interactions using the ENCORI open-source platform to predict these interactions from various sources, such as CLIP-seq, degradome-seq, and RNA-RNA interactome data [21]. Additionally, the database analyzes the correlation between lncRNA/pseudogene miRNAs and miRNA genes, with cutoff criteria of *R* < −0.1 and *p* < 0.05 set for gene pairs. For subsequent analysis, only miRNAs that emerged from more than two prediction programs were selected and from one pan-cancer dataset were considered.

## 3. Results

### 3.1. Pan-Cancer Gene Expression Analysis Data

**Figure 2:**
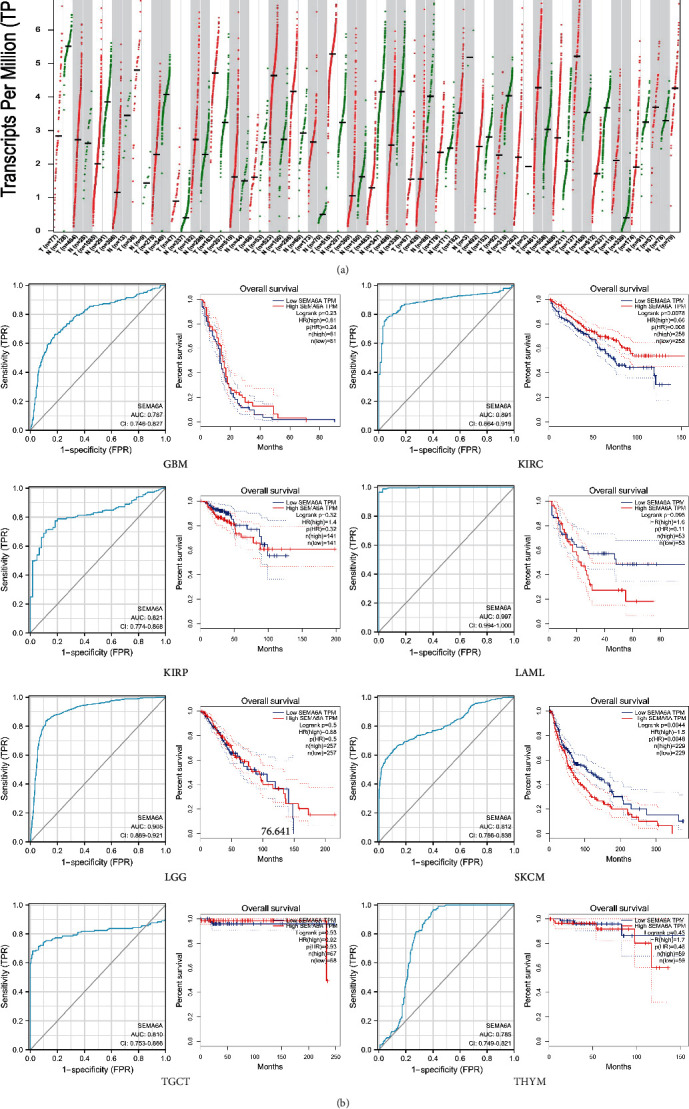
Gene expression analysis data in pan-cancer for SEMA6A. (a) Red font indicates a significant upregulation in tumor samples compared with normal samples, whereas green font indicates a significant downregulation in tumor samples compared with normal samples. (b) ROC and survival plots of SEMA6A in various cancer types from TCGA.

The SEMA6A expression level in pan-cancer was obtained from the GEPIA database, and it was revealed through analysis that SEMA6A expression level was higher in tumor tissues of various types of cancer, including glioblastoma multiforme, kidney renal clear cell carcinoma, kidney renal papillary cell carcinoma, acute myeloid leukemia, brain lower grade glioma, SKCM, testicular germ cell tumors, and thymoma as compared with normal tissues (Figure 1(a)). Furthermore, for evaluating the diagnostic value of SEMA6A, the addition of normal skin tissue from the GTEx dataset was carried out due to the lack of normal control specimens of SKCM in the TCGA dataset (Figure 2(a)). It was found that OS in the high-expression SEMA6A group was poor, and had a certain level of accuracy as indicated by the ROC curve (Figure 2(b)). However, a significant difference in OS was only observed for SKCM (HR > 1; *p* < 0.05).

### 3.2. Validation of SEMA6A Expression Level in Skin Melanoma Tissues and Cell Lines

**Figure 3:**
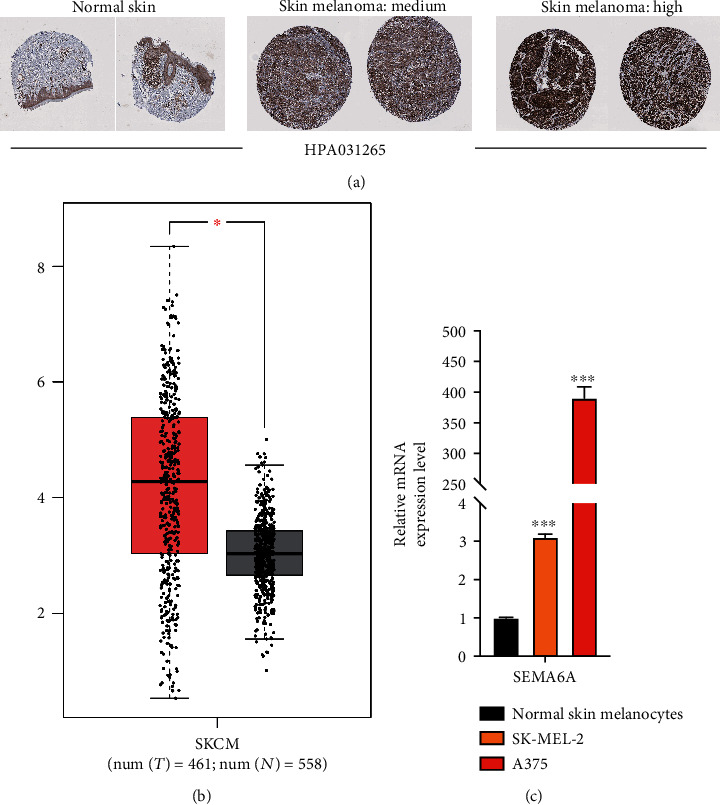
Representative immunohistochemistry images of SEMA6A in SKCM tissues and normal skin tissues (HPA) (a) SEMA6A expression level, ∗ represents *p* < 0.05 (b). The results of quantitative reverse transcription PCR (RT-qPCR) of SEMA6A expression level in SKCM cell lines (c) ∗∗∗, ∗∗, and ∗ represent significant differences compared with human primary normal skin melanocytes (*p* < 0.001, *p* < 0.01, and *p* < 0.05), respectively.

The SEMA6A expression level in SKCM tissues was investigated using the HPA. It was revealed that SEMA6A protein was not expressed in the normal skin group, whereas its high and medium expression levels were identified in SKCM tissues (Figure 3(a)). Additionally, a significant increase in SEMA6A expression level in SKCM tissues was noted compared with normal skin tissues obtained from the GTEx dataset in the GEPIA database (Figure 3(b)). To further validate the predictive value of SEMA6A expression level, this gene's expression level was evaluated in vitro. SEMA6A expression level in SKCM cell lines (A375 and SK-MEL-2) was significantly higher than that in the human primary normal skin melanocytes cell line (Figure 3(c)).

### 3.3. Co-Expression Analysis of SEMA6A in SKCM and GO and KEGG Pathway Enrichment Analyses

**Figure 4:**
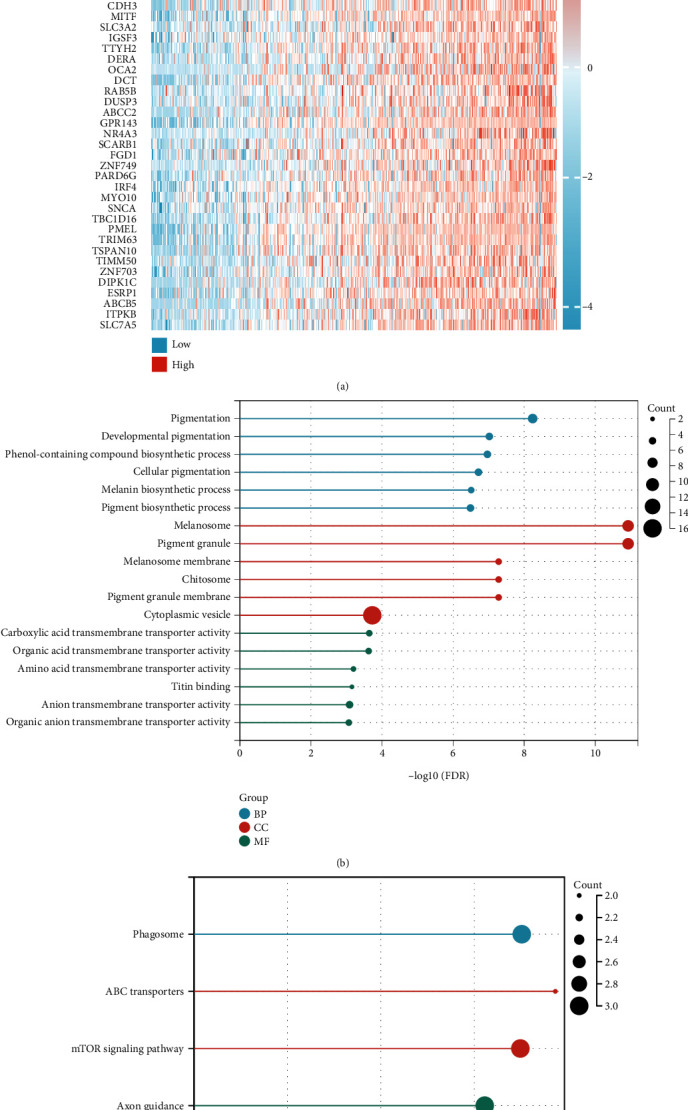
(a) The co-expression heatmap of the top 50 genes that were positively correlated with SEMA6A in SKCM. (b) The top 6 enriched GO functions included BP, CC, and MF for the co-expressed genes of SEMA6A. (c) The top 5 enriched KEGG pathway analyses for the co-expressed genes of SEMA6A.

The top 50 co-expressed genes that had a positive or a negative correlation with SEMA6A expression level in SKCM were found using a heatmap (Figure 4(a) and Supplementary Table 1). A total of 53 co-expressed genes were identified with Pearson's correlation coefficient >0.5 and *p* < 0.05. To better understand the functions of these genes, it was attempted to carry out the GO and KEGG pathway enrichment analyses using the Enrichr database. The GO and KEGG enrichment analyses were subsequently conducted on the co-expression genes (Figures 4(b) and 4(c)). The primary BP was found to be related to pigmentation, developmental pigmentation, biosynthesis of phenol-containing compounds, cellular pigmentation, and biosynthesis of melanin and pigment. The CC was mainly enriched in structures, such as melanosome, pigment granule, melanosome membrane, chitosome, pigment granule membrane, and cytoplasmic vesicle. In terms of MF, the genes were primarily involved in functions, such as carboxylic acid transmembrane transporter activity, organic acid transmembrane transporter activity, amino acid transmembrane transporter activity, titin binding, anion transmembrane transporter activity, and organic anion transmembrane transporter activity. The KEGG pathway enrichment analysis was mainly related to the phagosome, ATP-binding cassette (ABC) transporters, mammalian target of rapamycin (mTOR) signaling pathway, axon guidance, and bile secretion.

### 3.4. Association between SEMA6A Expression Level and Naive B Cells

**Figure 5:**
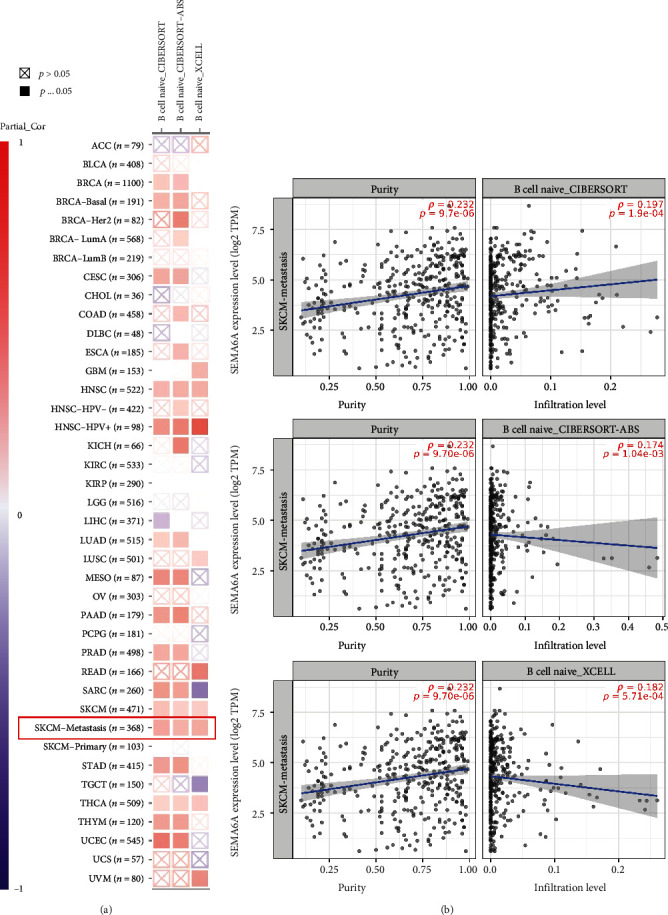
Correlation between SEMA6A expression level and infiltration of naive B cells in (a) pan-cancer and (b) SKCM-metastasis.

The crucial role in the initiation, progression, and metastasis of tumors is played by immune cells present in the tumor microenvironment [22, 23]. There has been increasing evidence highlighting the importance of tumor-associated fibroblasts and various immune cells in regulating tumor biological activities [24, 25]. SEMA6A expression level in SKCM-metastasis was investigated using CIBERSORT, CIBERSORT-ABS, and XCELL. The results of the analysis revealed a statistically significant positive correlation between SEMA6A expression level and estimated infiltration values of B cell naive (CIBERSORT: cor = 0.197, *p* = 1.9 × 10^−4^, CIBERSORT-ABS: cor = 0.174, *p* = 1.04 × 10^−3^, and XCELL: cor = 0.182, *p* = 5.71 × 10^−4^; Figures 5(a) and 5(b)).

### 3.5. hsa-miR-625-5p–SEMA6A Axis Was Identified in SKCM

**Figure 6:**
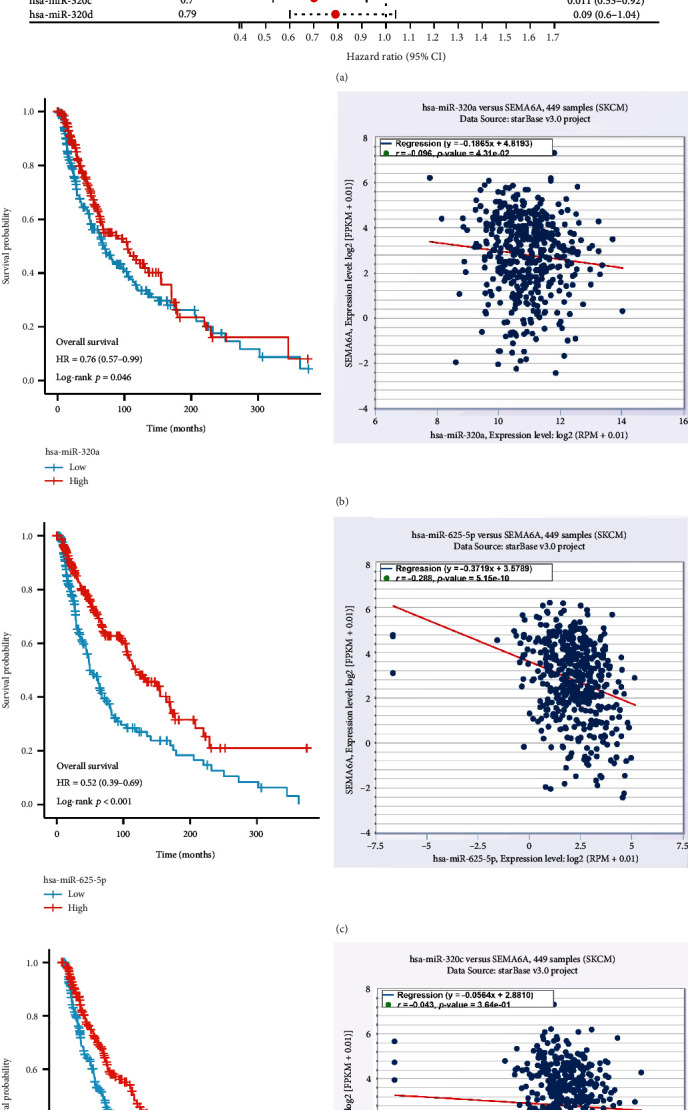
Identification of potential upstream miRNAs of SEMA6A and their prognostic values in SKCM. (a) Prognostic evaluation of upstream miRNAs of SEMA6A in the TCGA SKCM cohort based on OS. (b) Correlation analysis of hsa-miR-320a and SEMA6A expression levels with OS in SKCM. (c) Correlation analysis of hsa-miR-625-5p and SEMA6A expression levels with OS in SKCM. (d) Correlation analysis of hsa-miR-320c and SEMA6A expression levels with OS in SKCM.

miRNAs have been shown to play a significant role in various human BPs, including cancer development and occurrence, through mRNA binding [11, 26, 27]. To predict the upstream miRNAs of SEMA6A, the StarBase database (Table 1) was utilized, and 23 miRNAs were identified that could potentially target SEMA6A. The OS analysis of SKCM samples was conducted using the XIANTAO platform to improve visualization. As depicted in Figure 6(a), a longer OS was found for hsa-miR-320a, hsa-miR-625-5p, and hsa-miR-320c among all predicted miRNAs for SEMA6A in the high-risk group. In the subsequent analysis, three miRNA‑mRNA pairs were selected based on the ceRNA functional hypothesis, including hsa-miR-320a‑SEMA6A, hsa-miR-625-5p‑SEMA6A, and hsa-miR-320c‑SEMA6A. The correlation between SEMA6A and these miRNAs was further investigated, and it was revealed that only hsa-miR-625-5p exhibited a significant negative correlation with SEMA6A in SKCM (Figures 6(b), 6(c), and 6(d)).

### 3.6. Upstream Potential lncRNAs and Pseudogenes of hsa-miR-625-5p in SKCM

**Figure 7:**
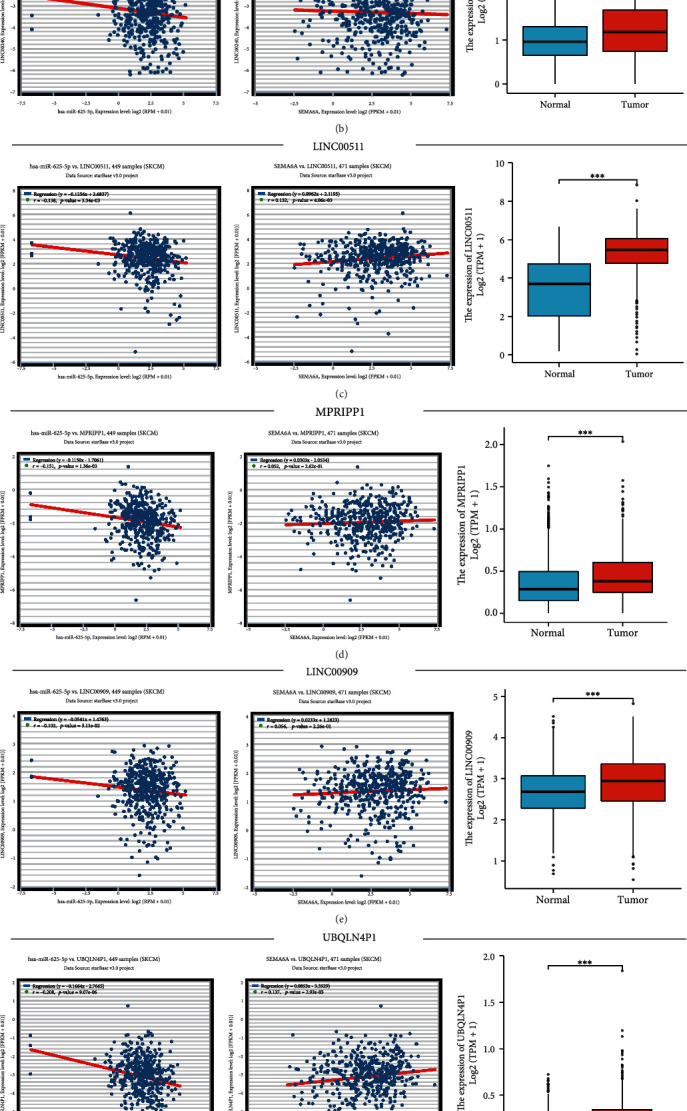
Exploration of upstream LncRNAs/pseudogenes of hsa-miR-625-5p in SKCM and their network. (a) Network diagram representing the upstream lncRNAs and pseudogenes of hsa-miR-625-5p in SKCM. The diamond shape represents lncRNAs, and the ellipse represents pseudogenes. (b) Analysis of expression levels and correlation of LINC00240 with SKCM. (c) Analysis of expression levels and correlation of LINC00511 with SKCM. (d) Analysis of expression levels and correlation of MPRIPP1 with SKCM. (e) Analysis of expression levels and correlation of LINC00909 with SKCM. (f) Analysis of expression levels and correlation of UBQLN4P1 with SKCM. (g) Analysis of expression levels and correlation of PVT1 with SKCM. (h) Results of RT-qPCR for LINC00511 expression levels in SKCM cell lines. (i) Results of RT-qPCR for UBQLN4P1 expression levels in SKCM cell lines. *Note*. ∗, ∗∗, and ∗∗∗ represent significant differences compared with human primary normal skin melanocytes (*p* < 0.05, p<0.01, and *p* < 0.001), respectively.

Pseudogenes and lncRNAs, two important isoforms of ncRNAs, may act as ceRNA by competing with mRNAs for shared miRNAs. To assess their potential to bind hsa-miR-625-5p, the StarBase database was used to predict upstream lncRNAs and pseudogenes. As shown in Figure 7(a) and Supplementary Table 2, 32 lncRNAs and 53 pseudogenes were obtained, respectively. Among them, 9 lncRNAs and 14 pseudogenes were found to be highly expressed in tumor tissues compared with normal tissues from TCGA and GTEx data controls. According to the ceRNA hypothesis, these lncRNAs and pseudogenes may act as oncogenes in SKCM. Correlation analysis indicated that hsa-miR-625-5p was significantly negatively correlated with four lncRNAs (LINC00240, PVT1, LINC00511, and LINC00909) and two pseudogenes (MPRIPP1 and UBQLN4P1). Furthermore, only one lncRNA (LINC00511) and one pseudogene (UBQLN4P1) were significantly positively correlated with SEMA6A (Figures 7(b), 7(c), 7(d), 7(e), 7(f), and 7(g); Table 2). These findings suggest that LINC00511 (lncRNA) and UBQLN4P1 (pseudogene) are the most likely upstream regulators of hsa-miR-625-5p in SKCM. The LINC00511 expression level in SKCM cell lines (A375 and SK-MEL-2) was significantly higher than that in the human primary normal skin melanocytes cell line (Figure 7(h)). However, the UBQLN4P1 gene was significantly under-expressed (Figure 7(i)).

## 4. Discussion

SKCM is a highly aggressive form of cancer that has caused significant morbidity and mortality worldwide. Despite the progress made in immunotherapy and targeted therapy, the incidence of recurrence, metastasis, and drug resistance remain a major challenge in treating this disease effectively. Therefore, it is crucial to discover new tumor markers that can aid in early diagnosis and improve treatment outcomes for patients with SKCM.

SEMA6A is a transmembrane-binding protein that has been extensively studied in neurological disorders, but its role in malignant tumors has only recently been investigated. Our study compared the expression of SEMA6A in melanomas using data from the TCGA and GTEx databases. By integrating clinical prognosis analysis with information from the HPA database, we discovered that SEMA6A shows promise as a predictor of outcomes and has significant potential for use in the diagnosis and treatment of SKCM.

The functional significance of co-expressed genes related to SEMA6A in SKCM was analyzed using the GO/KEGG method. Significant enrichment was observed in phagosomes, ABC transporters, and mTOR signaling pathways, which are associated with tumor autophagy, drug resistance, and apoptosis. Previous research has identified SEMA6A as a critical regulator of angiogenesis and endothelial cell survival [28], suggesting a potential role as a therapeutic target for reducing pathological angiogenesis [29]. Additionally, SEMA6A has been shown to be associated with tumor apoptosis in human oral cancer cell experiments [30]. These findings suggest that the SEMA6A gene may have a similar biological function in SKCM as observed in previous studies.

Immunotherapy has emerged as a significant area of research in the fight against malignant tumors, highlighting the growing importance of immunological research. One promising avenue for investigation is immune infiltration analysis, which has revealed a positive correlation between SEMA6A and B cell naive in metastatic SKCM. Recent studies have shed light on the complex role that B cells play in both promoting and inhibiting tumor growth. On one hand, B cells can directly destroy cancer cells through mechanisms, such as antibody-dependent cell cytotoxicity and phagocytosis, while also activating T cells to mount an immune response against tumors [30–32]. However, certain tumors, such as renal cell carcinoma and glioblastoma, exhibit a poor prognosis when infiltrated by B cells [33]. These contradictory findings suggest that different microenvironments or infiltration patterns may influence B cell differentiation and ultimately impact overall tumor outcomes. While the semaphorin family genes have been increasingly implicated in immune regulation, little is known about their specific role in cancer immunity. Further research is thus required to understand the immune response elicited by SEMA6A in SKCM [34].

Emerging research on biomarkers and therapeutic targets has highlighted the potential of ncRNAs, with miRNAs being a prominent type of ncRNA [35, 36]. These single-stranded molecules, which are typically 20–22 nucleotides long, can regulate gene expression post-transcriptionally [37]. As cancer progression is regulated by complex molecular mechanisms, an increasing number of studies have examined the role of miRNAs in this process [38, 39]. In the present study, 23 upstream miRNAs were identified that could potentially target SEMA6A, and after analyzing their correlation with expression and prognostic value (OS), it was found that hsa-miR-625-5p was the most promising candidate for binding to SEMA6A. This miRNA has been shown to be dysregulated in several types of cancer, including non-small cell lung cancer, cervical cancer, glioma, and gastric cancer, and also to be associated with malignant phenotypes (e.g., apoptosis and proliferation) [40–44].

LncRNAs and pseudogenes are two types of ncRNAs that can potentially act as ceRNAs by binding to miRNAs and indirectly regulating the expression of target mRNAs [9, 45]. The results of the present study and verification in cells showed that only LINC00511 exhibited a negative correlation with hsa-miR-625-5p and a positive correlation with SEMA6A, supporting the ceRNA hypothesis. Previous research has reported that LINC00511 could regulate trophoblast cell proliferation, apoptosis, invasion, and autophagy through the mir-31-5p/homeobox A7 axis [46], and promote temozolomide resistance in glioblastoma cells [47]. Additionally, a meta-analysis has revealed that increased LINC00511 expression level was associated with poor OS rates in multiple cancer types, suggesting its potential as an independent predictor for poor prognosis [48].

The results of the present research suggested that the Linc00511–hsa-miR-625-5p–SEMA6A axis could be associated with tumor autophagy, drug resistance, and cell apoptosis in SKCM.

## 5. Conclusions

In summary, it was revealed that the LINC00511–hsa-miR-625–5p-SEMA6A axis could regulate tumor autophagy, drug resistance, and apoptosis through phagosome, ABC transporters, and mTOR signaling pathways in SKCM, making it as a promising therapeutic target and a prognostic biomarker.

## Figures and Tables

**Table 1:** Prediction of miRNAs binding to SEMA6A.

Gene name	miRNA name	miRNA prediction databases		Prognostic values (OS)		
		Predicting databases	Number	HR	CI	Log-rank *p*
SEMA6A	hsa-miR-24-3p	PITA, microT, and TargetScan	3	1.2	0.91–1.58	0.187
SEMA6A	hsa-miR-27a-3p	PITA, microT, and miRmap	3	1.09	0.83–1.44	0.522
SEMA6A	hsa-miR-103a-3p	PITA, miRmap, TargetScan, and miRanda	4	1.1	0.84–1.45	0.495
SEMA6A	hsa-miR-107	PITA, miRmap,T argetScan, and miRanda	4	1.08	0.82–1.43	0.567
SEMA6A	hsa-miR-129-5p	PITA, microT, and miRmap	3	1.29	0.98–1.7	0.068
SEMA6A	hsa-miR-7-5p	PITA, microT, miRmap, and PicTar	4	1.04	0.79–1.36	0.801
SEMA6A	hsa-miR-218-5p	PITA, miRmap, PicTar, TargetScan, and miRanda	5	0.94	0.72–1.24	0.665
SEMA6A	hsa-miR-27b-3p	PITA, microT, miRmap, PicTar, TargetScan, and miRanda	6	1.18	0.9–1.55	0.24
SEMA6A	hsa-miR-128-3p	PITA, microT, PicTar, and TargetScan	4	1.16	0.88–1.53	0.286
SEMA6A	hsa-miR-138-5p	PITA, miRanda, and TargetScan	3	1.04	0.79–1.37	0.755
SEMA6A	hsa-miR-141-3p	PITA, microT, miRmap, PicTar, TargetScan, and miRanda	6	1.1	0.84–1.46	0.478
SEMA6A	hsa-miR-144-3p	PITA, microT, miRmap, TargetScan, and miRanda	5	1.17	0.89–0.54	0.254
SEMA6A	hsa-miR-320a	PITA, microT, and miRanda	3	0.76	0.57–0.99	0.046
SEMA6A	hsa-miR-200a-3p	PITA, microT, miRmap, PicTar, TargetScan, and miRanda	6	1.01	0.77–1.33	0.955
SEMA6A	hsa-miR-323a-3p	PITA, microT, and PicTar	3	1.21	0.92–1.59	0.176
SEMA6A	hsa-miR-338-3p	PITA, PicTar, and miRanda	3	1.19	0.9–1.57	0.2
SEMA6A	hsa-miR-491-5p	PITA, microT, miRmap, and miRanda	4	0.93	0.71–1.22	0.603
SEMA6A	hsa-miR-513a-5p	PITA, microT, miRmap, and PicTar	4	1.27	0.97–1.67	0.084
SEMA6A	hsa-miR-506-3p	PITA, microT, miRmap, PicTar, and miRanda	5	1.23	0.93–1.61	0.143
SEMA6A	hsa-miR-625-5p	PITA, microT, and miRmap	3	0.52	0.39–0.69	0.001
SEMA6A	hsa-miR-320b	PITA, microT, and miRanda	3	0.9	0.68–1.18	0.431
SEMA6A	hsa-miR-320c	PITA, microT, and miRanda	3	0.7	0.53–0.92	0.011
SEMA6A	hsa-miR-320d	PITA, microT, and miRanda	3	0.79	0.6–1.04	0.09

**Table 2:** Correlation of high expression of lncRNAs/pseudogenes with hsa-mir-625-5p and SEMA6A.

Name	Type	hsa-miR-625-5p		SEMA6A	
		Coefficient-*R*	*p*-Value	Coefficient-*R*	*p*-Value
LINC01132	lncRNA	0.123	8.91 × 10^−3^	−0.284	3.34 × 10^−10^
LINC01816	lncRNA	0.215	4.51 × 10^−6^	−0.103	2.56 × 10^−2^
LINC00240	lncRNA	−0.099	3.54 × 10^−2^	−0.033	4.70 × 10^−1^
PVT1	lncRNA	0.101	3.18 × 10^−2^	0.034	4.63 × 10^−1^
LINC00839	lncRNA	−0.055	2.47 × 10^−1^	−0.194	2.34 × 10^−5^
AC092368.3	lncRNA	0.015	7.57 × 10^−1^	−0.117	1.11 × 10^−2^
LINC00511	lncRNA	−0.138	3.34 × 10^−3^	0.132	4.06 × 10^−3^
LINC00909	lncRNA	−0.102	3.11 × 10^−2^	0.056	2.26 × 10^−1^
MIR503HG	lncRNA	0.044	3.48 × 10^−1^	−0.238	1.81 × 10^−7^
MTCO2P12	Pseudogene	0.044	3.55 × 10^−1^	0.147	1.38 × 10^−3^
AC098935.1	Pseudogene	0.004	9.29 × 10^−1^	0.13	4.79 × 10^−3^
MPRIPP1	Pseudogene	−0.151	1.36 × 10^−3^	0.052	2.62 × 10^−1^
PSMC1P1	Pseudogene	0.054	2.55 × 10^−1^	−0.043	3.48 × 10^−1^
UBQLN4P1	Pseudogene	−0.208	9.07 × 10^−6^	0.137	2.92 × 10^−3^
AMD1P3	Pseudogene	−0.042	3.72 × 10^−1^	0.095	4.02 × 10^−2^
LYPLA2P1	Pseudogene	−0.039	4.07 × 10^−1^	0.132	4.12 × 10^−3^
RNF216P1	Pseudogene	−0.002	9.62 × 10^−1^	0.158	5.58 × 10^−4^
GRK6P1	Pseudogene	0.16	6.58 × 10^−4^	−0.179	9.76 × 10^−5^
ZDHHC20P4	Pseudogene	−0.073	1.23 × 10^−1^	−0.104	2.36 × 10^−2^
AC111152.3	Pseudogene	0.154	1.04 × 10^−3^	−0.023	6.22 × 10^−1^
AC006504.3	Pseudogene	−0.016	7.38 × 10^−1^	0.032	4.88 × 10^−1^
RPL9P32	Pseudogene	−0.008	8.72 × 10^−1^	−0.022	6.33 × 10^−1^
PRR13P5	Pseudogene	0.149	1.49 × 10^−3^	0.068	1.38 × 10^−1^

## Data Availability

Data supporting this research article are available from the corresponding author or first author upon reasonable request.

## Supplementary Materials

Supplementary Materials Supplementary Table 1: Genes related to SEMA6A expression in SKCM (Pearson's correlation coefficient >0.5; *p* < 0.05).

Supplementary Materials Supplementary Table 2: Comparison of the expression levels of lncRNAs and pseudogenes that bind to hsa-miR-625-5p in SKCM tumor tissues and normal skin tissues.
