# Supplementary material for: Comprehensive Analysis to Identify LINC00511–hsa-miR-625-5p–SEMA6A Pathway Fuels Progression of Skin Cutaneous Melanoma
Source: Int J Genomics. 2023 Jul 3;2023:6422941. doi: 10.1155/2023/6422941 (PMC10332930; doi:10.1155/2023/6422941)
Supplement: Supplementary Materials — Supplementary Table 1: Genes related to SEMA6A expression in SKCM (Pearson's correlation coefficient >0.5; p < 0.05). [file 6422941.f1.pdf]

gene_name	gene_id	gene_biotype	cor_pearson	p_pearson	cor_spearman
DIPK1B	ENSG00000165716	protein_coding	0.575945255	1.05E-42	0.556153744
CAPN3	ENSG00000092529	protein_coding	0.503159679	2.11E-31	0.472579217
GCNT2	ENSG00000111846	protein_coding	0.500138593	5.45E-31	0.480528506
TUBB4A	ENSG00000104833	protein_coding	0.518146537	1.64E-33	0.459933432
PLXNC1	ENSG00000136040	protein_coding	0.508940919	3.33E-32	0.501185041
RASSF3	ENSG00000153179	protein_coding	0.54108361	6.02E-37	0.545387049
GREB1	ENSG00000196208	protein_coding	0.519353134	1.10E-33	0.477065106
CYP2U1	ENSG00000155016	protein_coding	0.54524625	1.34E-37	0.526226317
HPS4	ENSG00000100099	protein_coding	0.590220679	2.87E-45	0.572140474
PPM1H	ENSG00000111110	protein_coding	0.722599311	9.61E-77	0.713321526
CDK2	ENSG00000123374	protein_coding	0.684660223	5.29E-66	0.68235159
TBC1D7	ENSG00000145979	protein_coding	0.502077588	2.96E-31	0.480077435
TRPM1	ENSG00000134160	protein_coding	0.613792209	8.63E-50	0.615968314
RAB32	ENSG00000118508	protein_coding	0.535295194	4.69E-36	0.544204525
GMPR	ENSG00000137198	protein_coding	0.527514584	6.98E-35	0.534886923
HHAT	ENSG00000054392	protein_coding	0.587679579	8.40E-45	0.593691191
TRIM32	ENSG00000119401	protein_coding	0.503415898	1.94E-31	0.502792764
TFAP2A	ENSG00000137203	protein_coding	0.67361576	3.54E-63	0.695482514
MLANA	ENSG00000120215	protein_coding	0.569643006	1.30E-41	0.586401709
CDH3	ENSG00000062038	protein_coding	0.504872691	1.23E-31	0.474679922
MITF	ENSG00000187098	protein_coding	0.616413907	2.56E-50	0.617699207
SLC3A2	ENSG00000168003	protein_coding	0.511718569	1.36E-32	0.538219321
IGSF3	ENSG00000143061	protein_coding	0.516049887	3.29E-33	0.514364081
TTYH2	ENSG00000141540	protein_coding	0.579380724	2.61E-43	0.573728646
DERA	ENSG00000023697	protein_coding	0.542026717	4.29E-37	0.563002457
OCA2	ENSG00000104044	protein_coding	0.523001415	3.24E-34	0.502189429
DCT	ENSG00000080166	protein_coding	0.569806812	1.22E-41	0.53342343
RAB5B	ENSG00000111540	protein_coding	0.506908475	6.40E-32	0.518546641
DUSP3	ENSG00000108861	protein_coding	0.510589821	1.96E-32	0.531986747
ABCC2	ENSG00000023839	protein_coding	0.558957547	8.17E-40	0.564050351
GPR143	ENSG00000101850	protein_coding	0.611181213	2.85E-49	0.634600956
NR4A3	ENSG00000119508	protein_coding	0.541979145	4.36E-37	0.535037592
SCARB1	ENSG00000073060	protein_coding	0.538186043	1.69E-36	0.559984158
FGD1	ENSG00000102302	protein_coding	0.503425198	1.94E-31	0.493851541
ZNF749	ENSG00000186230	protein_coding	0.536161593	3.46E-36	0.53685124
PARD6G	ENSG00000178184	protein_coding	0.558426925	1.00E-39	0.555018048
IRF4	ENSG00000137265	protein_coding	0.554626848	4.20E-39	0.59185764
MYO10	ENSG00000145555	protein_coding	0.517210103	2.24E-33	0.510443992
SNCA	ENSG00000145335	protein_coding	0.506662964	6.92E-32	0.517174581
TBC1D16	ENSG00000167291	protein_coding	0.622628153	1.38E-51	0.624993956
PMEL	ENSG00000185664	protein_coding	0.652153613	5.02E-58	0.660957279
TRIM63	ENSG00000158022	protein_coding	0.62257741	1.41E-51	0.624085745
TSPAN10	ENSG00000182612	protein_coding	0.527580359	6.83E-35	0.505810477
TIMM50	ENSG00000105197	protein_coding	0.503062571	2.17E-31	0.524951191
ZNF703	ENSG00000183779	protein_coding	0.544097525	2.03E-37	0.522065766

DIPK1C	ENSG00000187773	protein_coding	0.543839853	2.23E-37	0.551527516
ESRP1	ENSG00000104413	protein_coding	0.543944654	2.15E-37	0.497016646
ABCB5	ENSG00000004846	protein_coding	0.582362143	7.68E-44	0.595937336
ITPKB	ENSG00000143772	protein_coding	0.545553778	1.20E-37	0.543579957
SLC7A5	ENSG00000103257	protein_coding	0.615646143	3.66E-50	0.63582305
CA14	ENSG00000118298	protein_coding	0.584784867	2.82E-44	0.566444667
CABLES1	ENSG00000134508	protein_coding	0.635532137	2.58E-54	0.630751108
CLCN7	ENSG00000103249	protein_coding	0.538693388	1.41E-36	0.538189819

p_spearman
0
0
0
0
0
0
0
0
0
0
0
0
3.15E-50
0
0
0
0
0
0
0
0
0
0
0
0
2.86E-31
0
0
0
0
0
0
0
0
0
0
0
0
0
0
0
6.89E-52
0
0
0

1.33E-38
0
2.49E-46
0
0
0
0
0
