# Supplementary material for: Comprehensive Analysis to Identify LINC00511–hsa-miR-625-5p–SEMA6A Pathway Fuels Progression of Skin Cutaneous Melanoma
Source: Int J Genomics. 2023 Jul 3;2023:6422941. doi: 10.1155/2023/6422941 (PMC10332930; doi:10.1155/2023/6422941)
Supplement: Supplementary Materials — Supplementary Table 2: Comparison of the expression levels of lncRNAs and pseudogenes that bind to hsa-miR-625-5p in SKCM tumor tissues and normal skin tissues. [file 6422941.f2.pdf]

lncRNA

| geneID | geneName | Tumor(J) vs Normal(I) (Wilcoxon rank sum test) | | |
|---|---|---|---|---|
| | | J-I | 95%CI | p-value |
| ENSG00000224259 | LINC01133 | -3.038 | -0.138 | < 0.001 |
| ENSG00000272205 | AL451050.2 | -0.485 | -0.162 | < 0.001 |
| ENSG00000229989 | MIR181A1HG | 0 | 0-0.014 | < 0.001 |
| ENSG00000227630 | LINC01132 | 0.056 | 0.043-0.057 | < 0.001 |
| ENSG00000231327 | LINC01816 | 0.549 | 0.494-0.605 | < 0.001 |
| ENSG00000236634 | AC016903.1 | ns | ns | ns |
| ENSG00000227308 | AC009502.1 | 0 | 0-0 | < 0.001 |
| ENSG00000251615 | AC104825.1 | -1.261 | -0.19 | < 0.001 |
| ENSG00000259959 | AC107068.1 | -0.585 | -0.079 | < 0.001 |
| ENSG00000224843 | LINC00240 | 0.195 | 0.127-0.264 | < 0.001 |
| ENSG00000273084 | AC092171.5 | -1.188 | -0.14 | < 0.001 |
| ENSG00000273014 | AC018645.2 | ns | ns | ns |
| ENSG00000272384 | AC016405.3 | -0.098 | -0.052 | < 0.001 |
| ENSG00000249859 | PVT1 | 0.377 | 0.248-0.508 | < 0.001 |
| ENSG00000233926 | AL591368.1 | 0 | 0-0 | < 0.001 |
| ENSG00000185904 | LINC00839 | 1.31 | 1.152-1.461 | < 0.001 |
| ENSG00000251381 | LINC00958 | -1.502 | -0.298 | < 0.001 |
| ENSG00000251562 | MALAT1 | -3.424 | -0.26 | < 0.001 |
| ENSG00000268836 | Z69706.1 | -0.714 | -0.157 | < 0.001 |
| ENSG00000261512 | AC092368.3 | 1.03 | 0.957-1.103 | < 0.001 |
| ENSG00000277559 | AC018553.1 | -0.637 | -0.115 | < 0.001 |
| ENSG00000261519 | AC010542.4 | -0.041 | -0.015 | < 0.001 |
| ENSG00000263126 | AC040162.3 | -0.916 | -0.12 | < 0.001 |
| ENSG00000271009 | AC116667.1 | -0.243 | -0.059 | < 0.001 |
| ENSG00000227036 | LINC00511 | 1.826 | 1.662-1.998 | < 0.001 |
| ENSG00000263731 | AC145207.5 | 0.059 | 0-0.121 | 0.062 |
| ENSG00000264247 | LINC00909 | 0.228 | 0.154-0.301 | < 0.001 |
| ENSG00000267100 | ILF3-DT | -0.499 | -0.169 | < 0.001 |
| ENSG00000232098 | AC012313.1 | -0.734 | -0.142 | < 0.001 |
| ENSG00000229807 | XIST | -0.472 | -0.153 | < 0.001 |
| ENSG00000230590 | FTX | -0.693 | -0.13 | < 0.001 |
| ENSG00000223749 | MIR503HG | 0.305 | 0.174-0.437 | < 0.001 |

pseudogene

| geneID | geneName | Tumor(J) vs Normal(I) (Wilcoxon rank sum test) | | |
|---|---|---|---|---|
| | | J-I | 95%CI | p-value |
| ENSG00000225972 | MTND1P23 | -1.111 | -0.228 | < 0.001 |
| ENSG00000229344 | MTCO2P12 | 0.775 | 0.695-0.854 | < 0.001 |
| ENSG00000248527 | MTATP6P1 | -0.811 | -0.183 | < 0.001 |
| ENSG00000238145 | AL731892.1 | 0 | -0.043 | < 0.001 |
| ENSG00000225154 | AL450996.1 | 0 | 0-0 | < 0.001 |
| ENSG00000235449 | AC098934.2 | -0.985 | -0.453 | < 0.001 |
| ENSG00000226945 | AC098935.1 | 0.137 | 0.115-0.138 | < 0.001 |

| | | | | |
|---|---|---|---|---|
| ENSG00000268412 | TRMT112P6 | -0.025 | -0.098 | < 0.001 |
| ENSG00000231362 | AC069271.1 | 0 | 0-0 | 0.239 |
| ENSG00000214820 | MPRIPP1 | 0.092 | 0.064-0.119 | < 0.001 |
| ENSG00000242849 | ALDOAP1 | 0 | 0-0 | 0.001 |
| ENSG00000214552 | COPS8P2 | 0 | -0.04 | < 0.001 |
| ENSG00000241506 | PSMC1P1 | 0.957 | 0.895-1.019 | < 0.001 |
| ENSG00000241627 | UBQLN4P1 | 0.027 | 0.013-0.047 | < 0.001 |
| ENSG00000249506 | ZEB2P1 | 0 | 0-0 | < 0.001 |
| ENSG00000249245 | NCOA4P3 | 0 | 0-0 | 0.003 |
| ENSG00000249286 | AMD1P3 | 0.075 | 0.057-0.084 | < 0.001 |
| ENSG00000270307 | MTATP6P2 | ns | ns | ns |
| ENSG00000220748 | AL032822.1 | 0 | 0-0 | 0.953 |
| ENSG00000228285 | LYPLA2P1 | 0.098 | 0.076-0.111 | < 0.001 |
| ENSG00000220483 | SLC25A51P1 | 0 | 0-0 | 0.683 |
| ENSG00000262095 | MTATP6P25 | ns | ns | ns |
| ENSG00000196204 | RNF216P1 | 0.555 | 0.493-0.617 | < 0.001 |
| ENSG00000224280 | AC005014.1 | 0 | 0-0 | 0.001 |
| ENSG00000233225 | SSBP3P1 | 0.014 | -0.067 | 0.396 |
| ENSG00000236692 | AC099654.3 | 0 | 0-0 | 0.629 |
| ENSG00000224415 | AC007683.1 | 0.013 | 0-0.039 | 0.132 |
| ENSG00000213590 | AL807752.1 | 0 | -0.014 | 0.364 |
| ENSG00000214297 | ALDOAP2 | 0 | 0-0 | 0.002 |
| ENSG00000254502 | AP003097.1 | 0 | 0-0 | < 0.001 |
| ENSG00000237493 | AC034102.1 | -0.016 | -0.115 | 0.594 |
| ENSG00000215571 | GRK6P1 | 0.028 | 0.014-0.029 | < 0.001 |
| ENSG00000230022 | FNTAP2 | 0 | 0-0 | < 0.001 |
| ENSG00000231417 | IRX1P1 | 0 | 0-0 | 0.261 |
| ENSG00000230793 | SMARCE1P5 | 0 | 0-0 | 0.763 |
| ENSG00000232380 | ZDHHC20P4 | 0.057 | 0.043-0.07 | < 0.001 |
| ENSG00000227105 | PARP1P1 | 0 | 0-0 | 0.031 |
| ENSG00000256385 | UBE2NP1 | 0 | 0-0 | < 0.001 |
| ENSG00000197358 | BNIP3P1 | -0.078 | -0.053 | < 0.001 |
| ENSG00000259045 | MTCO1P2 | 0 | 0-0 | < 0.001 |
| ENSG00000260174 | AC111152.3 | 0.057 | 0.043-0.057 | < 0.001 |
| ENSG00000258611 | YBX2P2 | -0.039 | -0.057 | < 0.001 |
| ENSG00000266994 | AC020663.1 | 0 | 0-0 | 0.04 |
| ENSG00000249176 | AC027801.3 | -0.057 | -0.098 | < 0.001 |
| ENSG00000267264 | AC006504.3 | 0.042 | 0-0.076 | 0.012 |
| ENSG00000242100 | RPL9P32 | 0.098 | 0.084-0.122 | < 0.001 |
| ENSG00000187534 | PRR13P5 | 0.29 | 0.233-0.345 | < 0.001 |
| ENSG00000268101 | CYP2G2P | 0 | 0-0 | < 0.001 |
| ENSG00000088340 | FER1L4 | -0.831 | -0.187 | < 0.001 |
| ENSG00000230870 | FBXW11P1 | -0.094 | -0.029 | < 0.001 |
| ENSG00000274602 | PI4KAP1 | -1.269 | -0.232 | < 0.001 |
| ENSG00000183506 | PI4KAP2 | -0.947 | -0.156 | < 0.001 |
| ENSG00000217835 | AL034397.1 | 0 | 0-0 | 0.966 |
